# Comparison of Selenium Nanoparticles and Sodium Selenite on the Alleviation of Early Atherosclerosis by Inhibiting Endothelial Dysfunction and Inflammation in Apolipoprotein E-Deficient Mice

**DOI:** 10.3390/ijms222111612

**Published:** 2021-10-27

**Authors:** Junying Xiao, Na Li, Shengze Xiao, Yuzhou Wu, Hongmei Liu

**Affiliations:** 1 Hubei Key Laboratory of Bioinorganic Chemistry and Materia Medica, School of Chemistry and Chemical Engineering, Huazhong University of Science and Technology, Wuhan 430074, China; junyingxiao21@hust.edu.cn (J.X.); m201870253@hust.edu.cn (N.L.); m201970247@hust.edu.cn (S.X.); wuyuzhou@hust.edu.cn (Y.W.); 2 Hubei Engineering Research Center for Biomaterials and Medical Protective Materials, Wuhan 430074, China

**Keywords:** sodium selenite, selenium nanoparticles, atherosclerosis, inflammation, endothelial dysfunction

## Abstract

Atherosclerosis and related cardiovascular diseases represent the greatest threats to human health, worldwide. Previous animal studies showed that selenium nanoparticles (SeNPs) and Na_2_SeO_3_ might have anti-atherosclerotic activity, but the underlying mechanisms are poorly elucidated. This study compared the anti-atherosclerotic activity of SeNPs stabilized with chitosan (CS-SeNPs) and Na_2_SeO_3_ and the related mechanism in a high-fat-diet-fed apolipoprotein E-deficient mouse model of atherosclerosis. The results showed that oral administration of both CS-SeNPs and Na_2_SeO_3_ (40 μg Se/kg/day) for 10 weeks significantly reduced atherosclerotic lesions in mouse aortae. Mechanistically, CS-SeNPs and Na_2_SeO_3_ not only alleviated vascular endothelial dysfunction, as evidenced by the increase of serum nitric oxide level and the decrease of aortic adhesion molecule expression, but also vascular inflammation, as evidenced by the decrease of macrophage recruitment as well as the expression of proinflammatory molecules. Importantly, these results were replicated within in-vivo experiments on the cultured human endothelial cell line EA.hy926. Overall, CS-SeNPs had a comparable effect with Na_2_SeO_3_ but might have more potential in atherosclerosis prevention due to its lower toxicity. Together, these results provide more insights into the mechanisms of selenium against atherosclerosis and further highlight the potential of selenium supplementation as a therapeutic strategy for atherosclerosis.

## 1. Introduction

Atherosclerotic cardiovascular diseases (CVD) are the leading cause of mortality and a major cause of morbidity and disability worldwide [1,2]. Atherosclerosis (AS) is a chronic inflammatory disease of the arterial wall, characterized by the formation of plaques consisting of foam cells, immune cells, vascular endothelial cells (ECs), vascular smooth muscle cells (VSMCs), platelets, extracellular matrix, and a lipid-rich core with extensive necrosis and fibrosis of surrounding tissues. Generally speaking, the development of atherosclerotic lesions contains three stages: initiation (a fatty streak lesion), lesion progression (a fibrous plaque) and complicated lesion (plaque rupture and thrombosis) [3,4]. A large number of studies have shown that the pathogenesis of AS includes hyperlipidemia, vascular endothelial dysfunction, inflammation and oxidative stress [4,5,6]. Besides, the increased immunogenicity of desialylated low-density lipoprotein (LDL) and mitochondrial mutations were also involved in the development of AS [7,8]. Among them, endothelial dysfunction and inflammation play pivotal roles in the initiation of AS. Normally, ECs play a crucial role in regulating vascular homeostasis, through their production of endogenous vasodilator nitric oxide (NO) and a functional barrier to resist the entry of the leukocytes streaming past them. When subjected to irritative stimuli (such as hyperlipidemia, oxidative stress, or pro-inflammatory mediators), ECs express adhesion molecules that capture leukocytes on their surfaces and chemokines that direct the migration of the bound leukocytes into the intima, which is named as endothelial dysfunction. Following endothelial dysfunction, monocytes are recruited from the blood into the intima, where they differentiate into macrophages and take up modified lipoproteins to become foam cells (the hallmark of early fatty streak lesions). Macrophages may also produce high levels of proinflammatory mediators, such as the cytokines interleukin-6 (IL-6) and tumor-necrosis factor-α (TNF-α), to induce VSMC migration from the middle layer of the artery wall into the intima. Intimal VSMCs may proliferate and take up modified lipoproteins, contributing to foam cell formation, and synthesize extracellular matrix components, thus generating a fibrous plaque. The concerted actions of activated ECs, VSMCs, monocyte-derived macrophages, and lymphocytes result in the production of a complex paracrine milieu of cytokines, growth factors, and reactive oxygen species within the vessel wall, which perpetuates a chronic proinflammatory state and fosters atherosclerotic lesion progression.

Selenium (Se), as an essential trace element, plays a vital role in human diseases and health [9]. In fact, a considerable number of observational studies from humans have shown a significant inverse association between Se concentration and CVD. Moreover, a benefit of Se supplementation in the prevention of CVD has been seen in population with low baseline Se status [10]. Evidence from animal studies shows consistent results that Se supplementation (mostly using Na_2_SeO_3_) could reduce atherosclerotic lesion formation in rabbits or apolipoprotein E-deficient (ApoE^-/-^) mice fed a high fat diet (HFD) [11,12,13,14]. However, the underlying mechanisms proposed in these animal studies have been limited to the protection of Se against oxidative stress, while other important risk events associated with disease progression, such as vascular endothelial dysfunction and inflammation, remain poorly elucidated.

It should be noted that the safety window for Se intake is fairly small, excess Se intake is toxic. In addition, the biological activity of Se is dependent upon its chemical form since different Se forms have different metabolic pathways [15]. As a unique form of Se, Se nanoparticles (SeNPs) exhibits higher bioavailability, stronger biological activity, and lower toxicity when compared with other Se forms such as selenious acid, Na_2_SeO_3_, selenomethionine (SeMet) and methylselenocysteine (SeMSC) [16,17,18]. For example, Na_2_SeO_3_, the most widely used Se form, exhibits excellent chemo-preventive and anticancer features [19], but SeNPs has been shown to be comparatively better as anticancer agent than Na_2_SeO_3_ [20]. Besides, many studies have demonstrated that SeNPs had stronger biological activities like antioxidant activity, antidiabetic activity, improving reproduction and antagonizing cadmium-induced cardiotoxicity than Na_2_SeO_3_ [21,22,23,24]. Therefore, SeNPs has been considered as a good candidate for replacement of other Se forms in clinical practice [17,25]. Our previous studies demonstrated that SeNPs had anti-atherosclerotic effect in ApoE^-/-^ mice after oral administration for 8 or 12 weeks, mainly by reducing hyperlipidemia and oxidative stress [26,27]. However, it still remains unknown whether SeNPs could alleviate endothelial dysfunction and inflammatory response. Moreover, it is quite necessary to explore whether the protective effect of SeNPs against AS is still superior to Na_2_SeO_3_.

The biological effect of nanoparticle is largely dependent on their surface properties and positively charged nanoparticles are taken up by cells at a faster rate [28]. Yu et al. reported that positive charge by chitosan surface decoration enhanced selective cellular uptake and anticancer efficacy of SeNPs [29]. Moreover, endothelial dysfunction and inflammation play pivotal roles in the initiation of AS. Hence, the current study investigated the effect of SeNPs stabilized with chitosan (CS-SeNPs) on inflammation and endothelial dysfunction in an early AS model of ApoE^-/-^ mice fed with HFD for 10 weeks and in human EC line EA.hy926. Besides, a comparison between CS-SeNPs and Na_2_SeO_3_ was performed. These studies will facilitate a better understanding of comparison effects of SeNPs and Na_2_SeO_3_ on alleviating atherosclerotic lesion and provide new insights into the mechanisms of Se against AS.

## 2. Results

### 2.1. Characterization of CS-SeNPs

**Figure 1:**
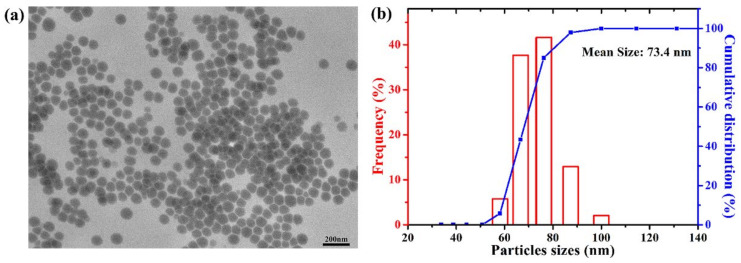
Characterization of CS-SeNPs. (**a**) TEM image; (**b**) DLS pattern.

The result of transmission electron microscope (TEM) showed that CS-SeNPs were uniformly spherical in shape and no significant aggregation was seen (Figure 1a). Dynamic light scattering (DLS) result showed that the mean size of CS-SeNPs was 73.4 nm (Figure 1b). Similar to our previous study [27,30], the infrared (IR) spectrum of CS-SeNPs was consistent with that of pure chitosan (Figure S1), indicating chitosan existed in the surface of SeNPs successfully. The zeta potential of CS-SeNPs was +32.1 mV. The positive surface charge of CS-SeNPs might mainly be attributed to NH_3_^+^ groups in chitosan. Besides, X-ray diffraction (XRD) pattern and X-ray photoelectron spectra (XPS) (data were not shown in this study) were consistent with our previous results [30], which indicated that the as-synthesized CS-SeNPs were amorphous and composed of element Se (0).

### 2.2. Both CS-SeNPs and Na_2_SeO_3_ Enhanced the Liver Se Contents in ApoE^-/-^ Mice

**Figure 2:**
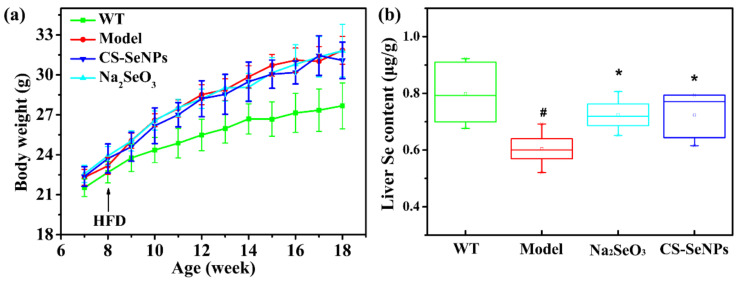
Effect of CS-SeNPs and Na_2_SeO_3_ on (**a**) body weight (*n* = 10) and (**b**) liver Se content (*n* = 6) in ApoE^-/-^ mice. The results were expressed as mean ± SD. ^#^ *p* < 0.05, compared with the WT group. * *p* < 0.05, compared with the AS model group.

The body weight and average food intake of all ApoE^-/-^ mice in this study was monitored every week. Compared to AS Model group, no significant difference was observed in the body weight (Figure 2a) and average food intake (Table S1) of CS-SeNPs and Na_2_SeO_3_ treated groups during the experimental period, suggesting that oral administration of CS-SeNPs and Na_2_SeO_3_ for 10 weeks did not affect the overall growth of ApoE^-/-^ mice. Besides, the liver Se content was detected to investigate the Se-supplement ability of CS-SeNPs and Na_2_SeO_3_ in ApoE^-/-^ mice. The liver Se contents in AS model group were significantly decreased compared with the WT group. As expected, the liver Se contents were significantly increased in both CS-SeNPs and Na_2_SeO_3_ treated groups, compared to AS Model group (Figure 2b), which demonstrated that oral administration of both CS-SeNPs and Na_2_SeO_3_ enhanced the Se retention in the liver. No significant difference of liver Se content was observed between CS-SeNPs and Na_2_SeO_3_ treated groups, suggesting that the Se-supplement ability of CS-SeNPs and Na_2_SeO_3_ was same.

### 2.3. Both CS-SeNPs and Na_2_SeO_3_ Alleviated The Atherosclerotic Lesions in ApoE^-/-^ Mice

**Figure 3:**
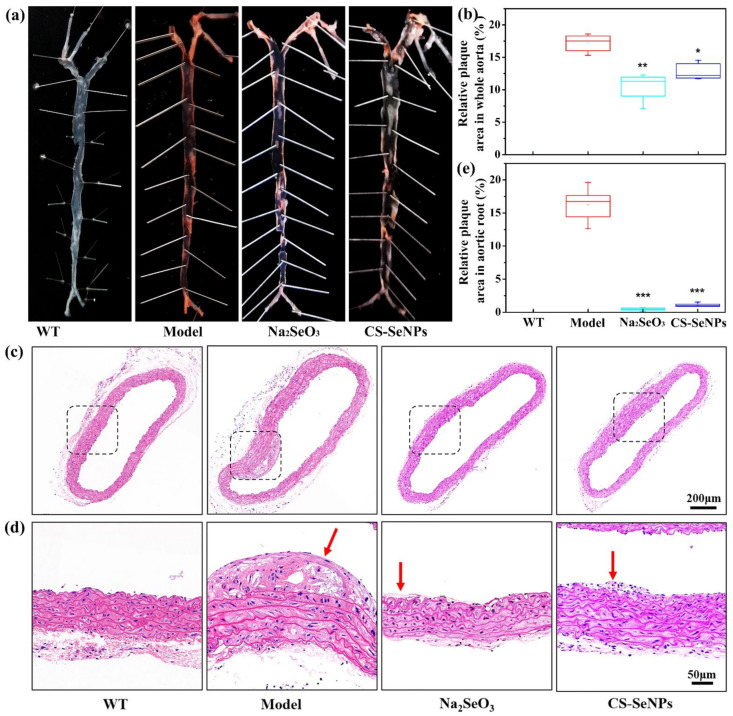
CS-SeNPs and Na_2_SeO_3_ reduced atherosclerotic lesions in HFD fed ApoE^-/-^ mice. (**a**) Whole aorta with *en face* Oil red O-staining. Representative images of Oil red O-stained whole aorta from each group were shown. (**b**) Atherosclerotic lesions were quantified and the values were expressed as percentage of lesion area (Oil red O-positive area) to total surface area of the aorta (*n* = 5). * *p* < 0.05, ** *p* < 0.01, compared with the AS model group. (**c**) Representative images of HE-stained aortic root cross section from each group (50×). (**d**) Magnified views (150×) corresponding to the black rectangles in (**c**). Red arrows refer to the atherosclerotic plaque. (**e**) Atherosclerotic lesions in aortic root were quantified and the values were expressed as percentage of lesion area to total surface area of the aortic root (*n* = 5). *** *p* < 0.001, compared with the AS model group.

To investigate the anti-atherosclerotic efficacy of CS-SeNPs and Na_2_SeO_3_, ApoE^-/-^ mice fed with HFD were supplemented with CS-SeNPs and Na_2_SeO_3_ at dose of 40 μg Se/kg body weight/day via an intragastric administration for 10 weeks. First, the severity of atherosclerotic lesions in whole aortas of ApoE^-/-^ mice was measured by Oil red O staining. In AS model group, significant atherosclerotic lesion with plaque occurred in whole aorta, and quantitative percentage of lesion area (Oil red O-positive area) was 17.1 ± 1.4% (Figure 3a,b), suggesting an early atherosclerosis was developed in ApoE^-/-^ mice fed with HFD for 10 weeks. Both CS-SeNPs and Na_2_SeO_3_ treatment significantly inhibited atherosclerotic lesion formation, and the lesions mainly occurred in the aortic arch (Figure 3a). Quantitative analysis showed that the lesion area was reduced 38.6% and 25.1% in Na_2_SeO_3_ and CS-SeNPs treated groups, respectively (Figure 3b).

Next, Hematoxylin-Eosin (HE) staining of aortic root cross section was performed in order to further confirm the effect of CS-SeNPs and Na_2_SeO_3_ on atherosclerotic lesion formation. As shown in Figure 3c, compared with WT group, the vessel wall of aortic root in AS model group was significantly thickened. And atherosclerotic plaques rich in lipids and foam cells could be obviously observed (Figure 3d). Compared with the AS model group, both CS-SeNPs and Na_2_SeO_3_ treatment significantly alleviated the vessel wall lesions. In the Na_2_SeO_3_ group, the vessel wall was almost normal and there were few plaques. In the CS-SeNPs group, the vessel wall was thickened, but few plaques were observed. Moreover, quantitative percentage of lesion area in aortic root was 16.2 ± 2.4% in AS model group, but very low in the Na_2_SeO_3_ group (0.43 ± 0.12%) and the CS-SeNPs group (1.14 ± 0.25%).

**Figure 4:**
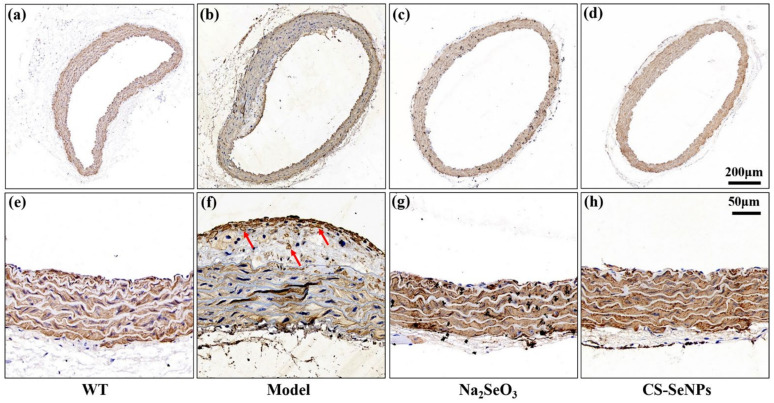
Immunohistochemical staining for α-SMA was performed to characterize the position of VSMCs in aortic root. (**a**–**d**) Representative images of immunohistochemical staining for α-SMA in aortic root (50×). (**e**–**h**) Magnified views (200×) corresponding to (**a**–**d**). Red arrows refer to brown α-SMA-positive cells.

Finally, because VSMC migration from the tunica media to the tunica intima is a hallmark of AS [5], immuno-histochemical staining for α smooth muscle actin (α-SMA), a maker for VSMCs, was performed to characterize the position of VSMCs in aortic root. In AS model group, a great deal of α-SMA-positive cells aggregated in the intima (Figure 4b), especially in the plaque (Figure 4f), suggesting the migration of VSMCs occurred. As comparison, all α-SMA-positive cells were found in the aortic media in CS-SeNPs and Na_2_SeO_3_ treated groups (Figure 4c,d,g,h). These results demonstrated that the treatment of both CS-SeNPs and Na_2_SeO_3_ inhibited VSMC migration.

Taken together, the above data indicated that CS-SeNPs and Na_2_SeO_3_ could alleviate atherosclerotic lesions in ApoE^-/-^ mice fed HFD. Moreover, the anti-atherosclerosis efficacy of CS-SeNPs was similar to that of Na_2_SeO_3_ in this experiment.

### 2.4. Both CS-SeNPs and Na_2_SeO_3_ Alleviated Vascular Endothelial Dysfunction in ApoE^-/-^ Mice

**Figure 5:**
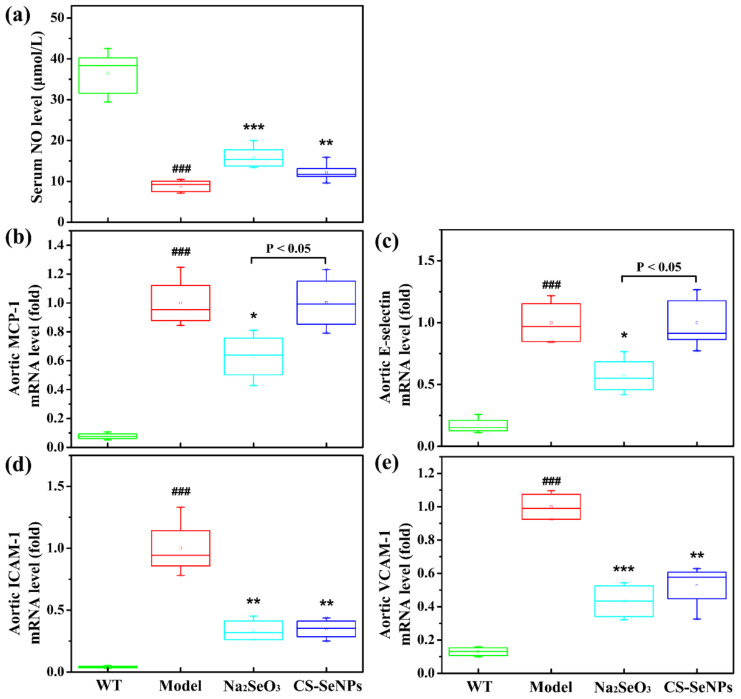
CS-SeNPs and Na_2_SeO_3_ attenuated endothelial dysfunction in ApoE^-/-^ mice. (**a**) Serum NO level (*n* = 10). (**b**–**e**) The mRNA expression levels of MCP-1 (**b**), E-selectin (**c**), ICAM-1 (**d**) and VCAM-1 (**f**) in the aorta determined by qPCR (*n* = 4–5). The results were expressed as mean ± SD. ^###^ *p* < 0.001, compared with the WT group. * *p* < 0.05, ** *p* < 0.01, *** *p* < 0.001, compared with the AS model group.

To investigate the mechanism by which CS-SeNPs and Na_2_SeO_3_ attenuated atherogenesis, we examined the effects of CS-SeNPs and Na_2_SeO_3_ on endothelial dysfunction, an initial step in vascular inflammation and atherogenesis. Endothelial dysfunction, broadly speaking, is not only characterized by a decrease of bioavailable NO, but also a promotion of monocyte adhesion molecule and chemokine expression [31]. As shown in Figure 5, in AS model group, the serum NO level was significantly decreased, while the expression of monocyte chemoattractant protein-1 (MCP-1), E-selectin, intercellular adhesion molecule-1 (ICAM-1) and vascular cell adhesion molecule-1 (VCAM-1) in the aorta were significantly increased compared with WT group, implied the endothelial dysfunction was occurred in ApoE^-/-^ mice induced by HFD. Compared with the AS model group, the serum NO level in CS-SeNPs- and Na_2_SeO_3_-treated group were significantly increased (Figure 5a). In addition, Na_2_SeO_3_ and CS-SeNPs both significantly reduced the expressions of ICAM-1 and VCAM-1 (Figure 5d,e). Differently, the expressions of MCP-1 and E-selectin were inhibited markedly by Na_2_SeO_3_ (Figure 5b,c), but not affected by CS-SeNPs. Taken together, these data indicate that both CS-SeNPs and Na_2_SeO_3_ could attenuate endothelial dysfunction, and the beneficial effect of Na_2_SeO_3_ was better than that of CS-SeNPs.

### 2.5. Both CS-SeNPs and Na_2_SeO_3_ Inhibited Macrophage Recruitment and the Inflammatory Response in ApoE^-/-^ Mice

Following endothelial dysfunction, adhesion molecules (e.g., VCAM-1, ICAM-1 and E-selectin) and chemokines (e.g., MCP-1) trigger the recruitment of monocytes to the aortic intima. Once in the intima, monocytes differentiate into tissue macrophages and engulf excess lipoprotein particles to become foam cells, subsequently leading to a cascade of inflammatory reactions and accelerating atherosclerotic lesion progression [3,6]. We next examined macrophage recruitment in the intima as well as the expression of inflammatory molecules in ApoE^-/-^ mice.

**Figure 6:**
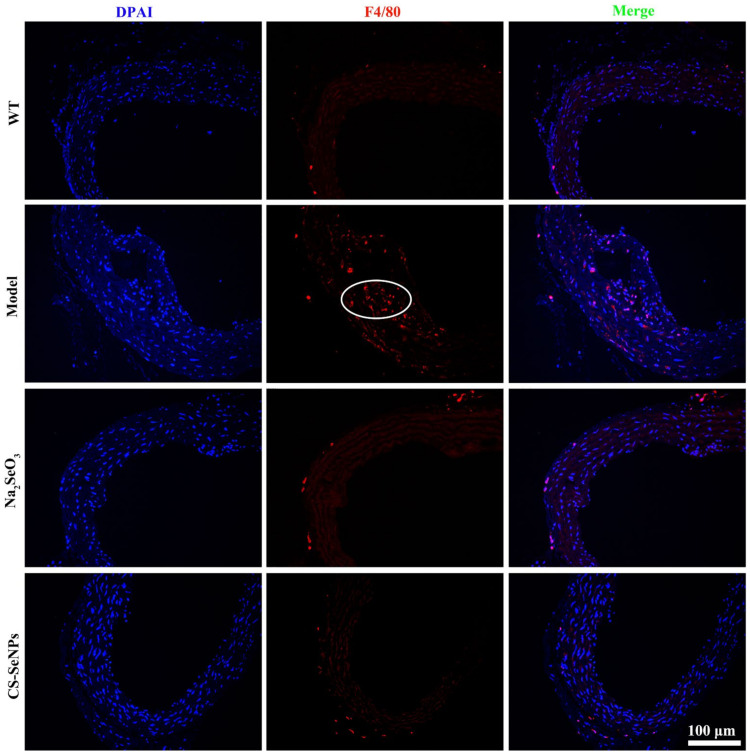
Immunofluorescence staining for F4/80 in aortic root tissue was performed to characterize macrophages in the aortic root. Representative images of immunofluorescence staining for F4/80 (red) in aortic root tissue (200×) are shown. White circles refer to F4/80-positive macrophages in plaque.

F4/80 is a widely used marker for monocytes and many tissue macrophages [32]. As assessed by immuno-histochemistry staining for F4/80 expression, many F4/80-positive cells were observed in the aortic root of AS model group, suggesting macrophage recruitment in the aortic intima. In comparison, no obvious F4/80-positive cells was observed in both the CS-SeNPs- and Na_2_SeO_3_-treated groups (Figure 6).

**Figure 7:**
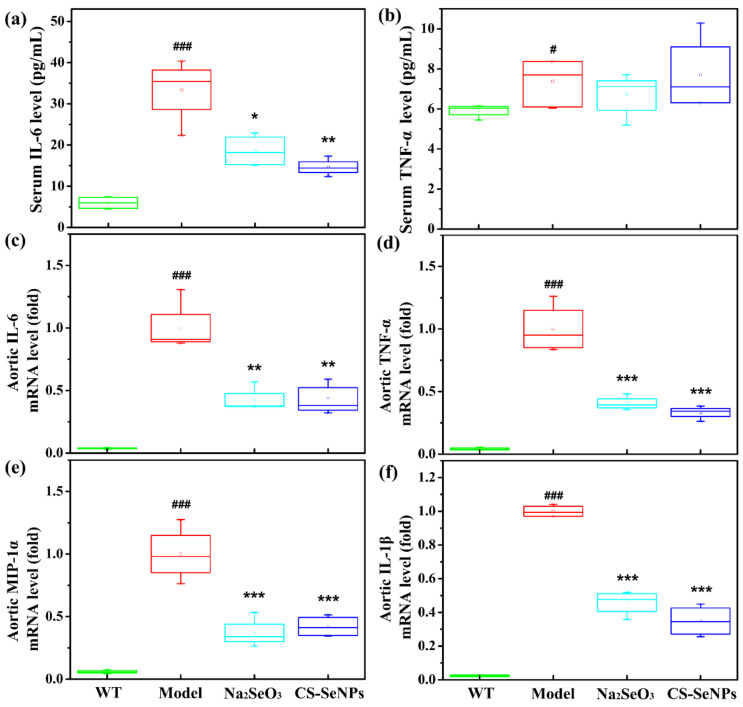
CS-SeNPs and Na_2_SeO_3_ inhibited inflammatory response in serum and the aorta. (**a**,**b**) The levels of serum IL-6 and TNF-α, assayed by ELISA (*n* = 8–10); (**c**–**f**) the mRNA expression levels of IL-6 (**c**), TNF-α (**d**), MIP-1α (**e**) and IL-1β (**f**) in the aorta, assayed by qPCR (*n* = 4–5). The results were expressed as mean ± SD. ^#^ *p* < 0.05, ^###^ *p* < 0.001, compared with the WT group. * *p* < 0.05, ** *p* < 0.01, *** *p* < 0.001, compared with the AS model group.

The results of ELISA analysis showed a remarkable increase of serum IL-6 level in AS model group compared with the WT group (Figure 7a). Compared with AS model group, serum IL-6 level was markedly reduced, by 56.3% and 44.3% in the Na_2_SeO_3-_ and CS-SeNPs-treated groups, respectively (Figure 7a). However, no significant difference was found in serum TNF-α level among all groups (Figure 7b).

The expression of inflammatory molecules IL-6, TNF-α, macrophage inflammatory ptotein-1α (MIP-1α) and interleukin-1β (IL-1β) in the aorta were assessed by real-time quantitative polymerase chain reaction (qPCR). Compared with the WT group, the expressions of these inflammatory molecules in the aorta were increased significantly in the AS model group (Figure 7c–f), suggesting the inflammatory response was occurred in the aorta. Excitingly, compared with the AS model group, the expressions of IL-6, TNF-α, MIP-1α and IL-1β were all significantly decreased in both the Na_2_SeO_3-_ and CS-SeNPs-treated groups.

### 2.6. CS-SeNPs and Na_2_SeO_3_ Inhibited Oxidative Stress in ApoE^-/-^ Mice

Oxidative stress occurs when the production of oxidants outweighs an organism’s antioxidant capabilities, resulting in cellular/tissue damage [33]. Malondialdehyde (MDA), an end product of lipid peroxidation, was often used as a biomarker of oxidative stress [34]. Total antioxidant capacity (T-AOC) was determined to find out the total enzymatic and non-enzymatic antioxidant activity. Glutathione peroxidase (GPx), the most abundant selenoprotein in mammals, is an intracellular antioxidant enzyme by reducing H_2_O_2_ to water and lipid hydroperoxides to their corresponding alcohols [35,36]. Compared with WT group, serum MDA level was significantly increased, while T-AOC and GPx activity were significantly decreased in AS model group (Table 1), indicating the increase of oxidative stress. Na_2_SeO_3_ significantly increased serum T-AOC and GPx activity and reduced serum MDA level, demonstrating excellent antioxidant activity. CS-SeNPs treatment significantly increased serum T-AOC and GPx activity, though had no significant effect on serum MDA level.

### 2.7. Both CS-SeNPs and Na_2_SeO_3_ Inhibited Lipopolysaccharide (LPS)-Induced Endothelial Dysfunction and Inflammatory Response in Human ECs

In order to further confirm the effect of CS-SeNPs and Na_2_SeO_3_ on endothelial dysfunction and inflammation, in-vitro-cultured human EC line EA.hy926 was used. The effect of CS-SeNPs and Na_2_SeO_3_ on the viability of ECs was first measured using MTT assay. As presented in Figure S2, the cell viability had no change after treatment with 0.5, 1 and 2 μM CS-SeNPs alone for 24 h but was significantly reduced after treatment with the same doses of Na_2_SeO_3_. These data suggested that CS-SeNPs at the range of 0.5–2 μM was relatively safe to cells under our experimental condition, but Na_2_SeO_3_ at the same range of dose was toxic. Thus, in the following experiments, 0.5 μM CS-SeNPs or Na_2_SeO_3_ was used to test their positive role against LPS-induced effects.

**Figure 8:**
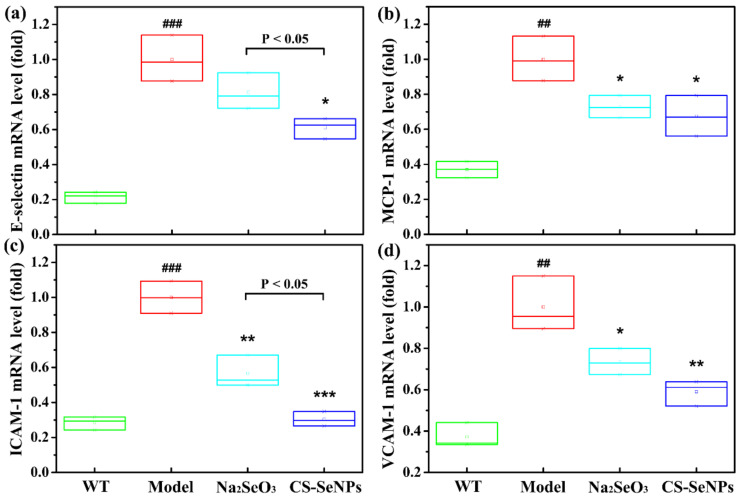
CS-SeNPs and Na_2_SeO_3_ attenuated LPS-induced endothelial dysfunction in ECs. Cells were induced with 1 μg/mL LPS for 12 h alone or pretreated with 0.5 μM CS-SeNPs or Na_2_SeO_3_ for 12 h before exposure to LPS. The mRNA expression levels of E-selectin (**a**) MCP-1 (**b**) ICAM-1 (**c**) and VCAM-1 (**d**) were assayed by qPCR. The results were expressed as mean ± SD (*n* = 3). ^##^ *p* < 0.01, ^###^ *p* < 0.001, compared with control cells (Con). * *p* < 0.05, ** *p* < 0.01, *** *p* < 0.001, compared with cells treated with LPS alone.

The results of qPCR showed the expressions of adhesion molecules and chemokines, including E-selectin, MCP-1, ICAM and VCAM, were significantly increased in LPS-stimulated ECs (Figure 8), indicating that endothelial dysfunction was induced by LPS in cells. Pretreatment with CS-SeNPs and Na_2_SeO_3_ significantly attenuated the expression of MCP-1, ICAM and VCAM induced by LPS. Differently, CS-SeNPs pretreatment significantly inhibited the induction of E-selectin expression by LPS, but Na_2_SeO_3_ pretreatment had no effect. In addition, the inhibitory effect of CS-SeNPs on ICAM expression was stronger than Na_2_SeO_3_ (*p* < 0.05).

**Figure 9:**
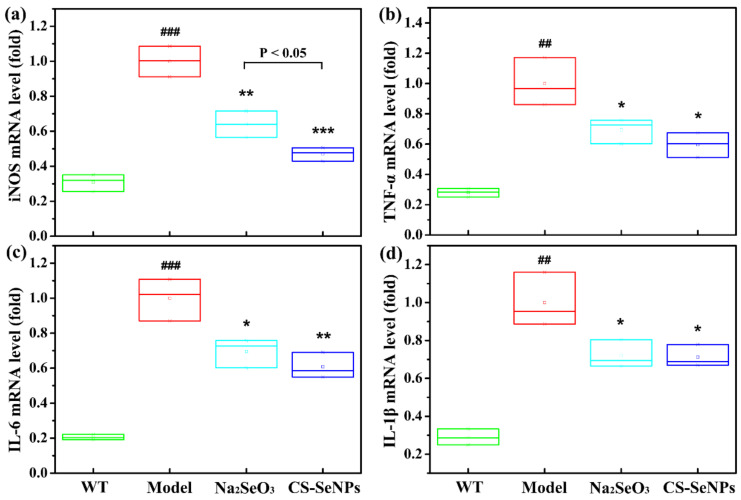
CS-SeNPs and Na_2_SeO_3_ inhibited the inflammatory response induced by LPS in ECs. Cells were induced with 1 μg/mL LPS for 12 h alone or pretreated with 0.5 μM CS-SeNPs or Na_2_SeO_3_ for 12 h before exposure to LPS. The mRNA expression levels of iNOS (**a**), TNF-α (**b**), IL-6 (**c**) and IL-1β (**d**) were assayed by qPCR. The results were ex-pressed as mean ± SD (*n* = 3). ^##^ *p* < 0.01, ^###^ *p* < 0.001, compared with control cells (Con). * *p* < 0.05, ** *p* < 0.01, *** *p* < 0.001, compared with cells treated with LPS alone.

The activation of inducible nitric oxide synthase (iNOS) enhances inflammatory processes within the vascular wall and contributes to AS progression [37]. As shown in Figure 9a, qPCR result showed that LPS induced the expression of iNOS in ECs, which was significantly inhibited by the pretreatment of CS-SeNPs and Na_2_SeO_3_. By comparison, CS-SeNPs exhibited a better effect than Na_2_SeO_3_ (*p* < 0.05). Furthermore, both CS-SeNPs and Na_2_SeO_3_ pre-treatment significantly suppressed the expression of proinflammation cytokines (TNF-α, IL-6 and IL-1β) induced by LPS in ECs (Figure 9b–d). Taken together, the above data indicate that pretreatment with CS-SeNPs and Na_2_SeO_3_ could inhibit LPS-induced endothelial dysfunction and inflammation in in-vitro-cultured ECs, which is consistent with the results from ApoE^-/-^ mice.

### 2.8. Both CS-SeNPs and Na_2_SeO_3_ Inhibited the Activation of Nuclear Factor-κB (NF-κB) Signaling Pathway Induced by LPS in Human ECs

**Figure 10:**
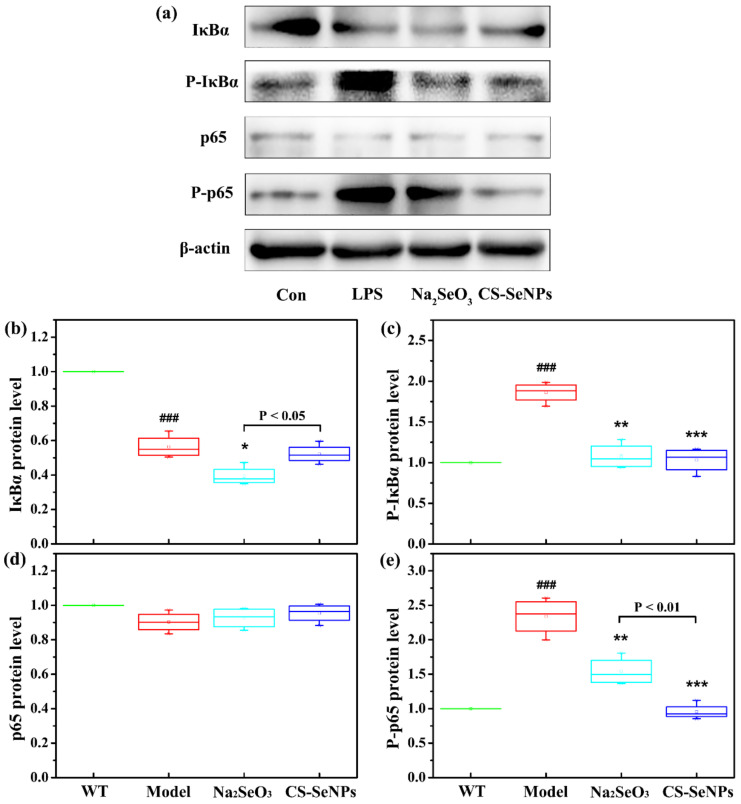
CS-SeNPs and Na_2_SeO_3_ suppressed LPS-induced activation of NF-κB signaling pathway in ECs. After pretreatment with or without 0.5 μM CS-SeNPs or Na_2_SeO_3_ for 12 h, cells were stimulated with LPS for another 12 h. Western blot analysis was done to determine the protein levels of IκBα, phosphorylated IκBα (P-IκBα), p65, phosphorylated p65 (Ser 536) (P-p65) and β-actin. (**a**) Representative Western blot bands. (**b**–**e**) Quantification analysis of IκBα (**b**), P-IκBα (**c**), p65 (**d**) and P-p65 (**e**) normalized to β-actin. The results were expressed as mean ± SD (*n* = 4). ^###^ *p* < 0.001, compared with the control cells (Con). * *p* < 0.05, ** *p* < 0.01, *** *p* < 0.001, compared with cells treated with LPS alone.

As the NF-κB signaling pathway plays a central role in the proinflammatory activation of endothelium in atherogenesis [31], the activation of the NF-κB signaling pathway in LPS-stimulated ECs and the effect of CS-SeNPs and Na_2_SeO_3_ were next examined. Western blot was used to evaluate the phosphorylation and degradation of IκBα, a classical pathway of NF-κB activation, and the phosphorylation of NF-κB p65 (Ser536), an alternative pathway of NF-κB activation. Compared with the control cells, LPS reduced the protein level of IκBα and simultaneously increased the phosphorylated levels of IκBα (P-IκBα) and p65 (P-p65) (Figure 10), indicating both classical pathway and alternative pathway of NF-κB signaling was activated. Compared with cells treated with LPS alone, though no significant difference was observed in the protein level of IκBα, the pretreatment of CS-SeNPs and Na_2_SeO_3_ significantly inhibited the phosphorylation of IκBα and p65 induced by LPS, indicating they could inhibit LPS-induced activation of NF-κB signaling pathway. Comparatively, CS-SeNPs exhibited a stronger inhibitory effect on the phosphorylation of p65 than Na_2_SeO_3_ (*p* < 0.01).

## 3. Discussion

Previous animal studies from other laboratories and ours showed that Na_2_SeO_3_ and SeNPs might prevent experimental AS in HFD-fed rabbits or ApoE^-/-^, mice mainly by inhibiting oxidative stress [11,12,13,14,26,27]. However, it remains unknown whether endothelial dysfunction and inflammation, another two important risk factors of AS, were involved in their anti-atherosclerotic effect. The present study showed that CS-SeNPs and Na_2_SeO_3_ (at the dose of 40 μg Se/kg body weight/day) could alleviate HFD-induced early atherosclerotic lesions in ApoE^-/-^ mice after oral administration for 10 weeks. More importantly, we found that the anti-atherosclerotic effect of CS-SeNPs or Na_2_SeO_3_ was accompanied by the alleviation of endothelial dysfunction and inflammation. Additionally, in-vitro studies showed that the pretreatment of both CS-SeNPs and Na_2_SeO_3_ significantly inhibited LPS-induced endothelial dysfunction, inflammation and activation of NF-κB signaling pathway in human EC line EA.hy926. These results provide more insights into the mechanisms of Se against AS and further highlight the potential beneficial effect of Se supplementation as a therapeutic strategy for AS.

Endothelial dysfunction is referred as an important contributor to the pathobiology of AS [31,38]. In this work, endothelial dysfunction was induced in HFD fed ApoE^-/-^ mice, as demonstrated by the decrease of serum NO level as well as the increase of the adhesion molecule (E-selectin, ICAM-1 and VCAM-1) and chemokine (MCP-1) expression in the aorta. CS-SeNPs and Na_2_SeO_3_ not only reversed the decrease of serum NO level, but also the increase of ICAM-1 and VCAM-1 expression (Figure 5). Moreover, Na_2_SeO_3_ significantly reduced the expression of MCP-1 and E-selectin. These results fully illustrated that CS-SeNPs and Na_2_SeO_3_ might alleviate AS in HFD-fed ApoE^-/-^ mice by inhibiting endothelial dysfunction.

A unifying view of the pathophysiology of AS proposes that inflammation has a key role and transduces the effects of many known risk factors for the disease. Inflammatory signaling alters the behavior of the intrinsic cells (ECs and VSMCs) of the artery wall, and recruits further inflammatory cells (such as monocyte-derived macrophages, T cells) that interact to promote lesion formation and complications [5]. In this study, CS-SeNPs and Na_2_SeO_3_ inhibited several hallmarks of inflammation, including macrophage recruitment, VSMC migration, the increased serum IL-6 level, and the elevated expression of aortic TNF-α, MIP-1α, IL-6 and IL-1β in HFD fed ApoE^-/-^ mice (Figure 4, Figure 6 and Figure 7). These results indicated the anti-inflammatory property might be involved in CS-SeNPs and Na_2_SeO_3_ modulation of AS.

In this study, the inhibition of CS-SeNPs and Na_2_SeO_3_ on endothelial dysfunction and inflammation was further evaluated using LPS-stimulated human EC line EA.hy926. Much evidence has shown that the exposure of ECs to LPS, a Gram-negative bacterial product, can result in endothelial dysfunction (the increased expression of adhesion molecules and chemokines) and inflammatory response (the increased expression of iNOS and pro-inflammatory cytokines) through the activation of NF-κB signaling pathway [39,40,41]. In present study, endothelial dysfunction (as demonstrated by the increased expression of MCP-1, E-selectin, ICAM-1 and VCAM-1), the inflammatory response (as demonstrated by the elevated expressions of iNOS, TNF-α, IL-6 and IL-1β) and the activation of the NF-kB signaling pathway were also induced by LPS in EA.hy926 cells (Figure 8, Figure 9 and Figure 10). Excitingly, pretreatment with CS-SeNPs and Na_2_SeO_3_ could inhibit all these undesirable effects of LPS, proving again the inhibitory effect of CS-SeNPs and Na_2_SeO_3_ on endothelial dysfunction and inflammation.

The potential of CS-SeNPs and Na_2_SeO_3_ to improve endothelial dysfunction or inflammation might be due to their antioxidant and redox-regulating properties, conferred by certain selenoproteins. First, CS-SeNPs and Na_2_SeO_3_ might combat oxidative stress through their antioxidant activities directly or indirectly. Plenty of evidence from data of laboratory and clinical studies suggests that oxidative stress plays a pivotal role in the pathogenesis of AS. Oxidative stress not only promotes oxidative modification of lipids and proteins (particularly the oxidative modification of lipoproteins), but also contributes to endothelial dysfunction, the activation of macrophage and the formation of foam cells, resulting in the aggravation of the AS [38,42]. The most well-characterized function of Se is its ability to mitigate oxidative stress through antioxidant-functioning selenoproteins, including the well-studied GPx and thioredoxin reductase (TrxR) families. In this study, supplementation of CS-SeNPs and Na_2_SeO_3_ enhanced the Se retention in the liver of (Figure 2b), implying that they were absorbed into the body. Moreover, both CS-SeNPs and Na_2_SeO_3_ significantly increased GPx activity and T-AOC and thus inhibited oxidative stress (Table 1). Secondly, CS-SeNPs and Na_2_SeO_3_ might protect against endothelial dysfunction and inflammation through redox regulation of inflammatory signaling pathways (such as NF-κB, mitogen-activated protein kinase) that lead to cytokine/chemokine production. For example, NF-κB is a pivotal transcriptional factor that regulates the expression of many pro-inflammatory cytokines, chemokines, adhesion molecules in cells [43,44]. The inhibition of NF-κB activation could inhibit endothelial dysfunction, vascular inflammation and the formation of AS [45]. Many studies had reported Na_2_SeO_3_ supplementation (at the range of 0.5–5 μM) could decrease the expression of pro-inflammatory gene and adhesion molecule by suppressing NF-κB activation [46,47,48,49]. Additionally, Zhu et al. reported SeNPs (0.5 μM), decorated with Ulva lactuca polysaccharide, could attenuate colitis by inhibiting NF-κB-mediated hyper inflammation [50]. In this study, we found the degradation and phosphorylation of IκBα as well as the phosphorylation of NF-κB p65 were increased in LPS-stimulated EC line EA.hy926, indicating the activation of NF-κB signaling pathway. The pretreatment with CS-SeNPs or Na_2_SeO_3_ inhibited LPS-induced NF-κB activation, as evidenced by the significant reduce of the phosphorylation of IκBα and NF-κB p65 (Figure 10).

Though Na_2_SeO_3_ is the most widely used Se supplement, it exhibits a narrow margin between beneficial and toxic effects. Many studies have shown that SeNPs has less toxicity than Na_2_SeO_3_ [51,52]. The lower toxicity of CS-SeNPs was also confirmed in our in vitro experiments on cultured human EC line EA.hy926. CS-SeNPs at the range of 0.5–2 μM had no effect on EC viability, but Na_2_SeO_3_ at the same range of dose significantly decreased cell viability, exhibiting significant toxicity (Figure S1). Furthermore, we also observed that CS-SeNPs was more efficient than Na_2_SeO_3_ in suppressing LPS-induced E-selectin, ICAM-1 and iNOS expression as well as NF-κB p65 phosphorylation in ECs. These results suggested that SeNPs might have a greater potential to serve as a therapeutic agent for AS than Na_2_SeO_3,_ considering its low toxicity.

It should be noted that, though our previous work had shown that oral administration of CS-SeNPs for 12 weeks inhibited the formation of AS, based on the same detection methods used in this work (including Oil red O staining, HE staining, the level of NO and MDA in serum, serum GPx activity), this work is remarkably novel in two respects. First, our previous study demonstrated that anti-atherosclerotic activity of CS-SeNPs might attribute to its inhibition of oxidative stress and hyperlipidemia [26,27], but this work focused on the inhibitory effect of CS-SeNPs on endothelial dysfunction (especially the expression of adhesion molecules and chemokines) and inflammation (including macrophage recruitment in the intima and the expression of inflammatory molecules in serum and aorta). Secondly, the effect of CS-SeNPs on LPS-induced endothelial dysfunction, inflammatory response and activation of NF-κB signaling pathway was evaluated using the in-vitro-cultured human EC line, EA.hy926, in order to further confirm the results from animal experiments.

However, there are still some loopholes to be answered by the present study. Firstly, chitosan was used as a stabilizer or capping agent to control SeNPs size and maintain SeNPs stabilization in aqueous solution. Previous studies reported that chitosan also had anti-atherosclerotic activity in an ApoE^-/-^ mouse model of AS [53,54]. Thus, whether the anti-atherosclerotic activity of CS-SeNPs is attributed to chitosan or SeNPs—or the combination of the two—needs to be clarified. However, our previous study showed the amount of chitosan in as-synthesized CS-SeNPs was very low (only 0.13%) [30]. We think that such a low level of chitosan present in CS-SeNPs would have no effect, independent from Se, on AS development. Secondly, recent studies have showed that desialylation of LDL and mitochondrial mutation also play important roles in AS development. The ability of Se to stimulate antioxidant activity in cells could protect cells from generation of mitochondrial mutation. Therefore, further studies aimed at addressing the effect of Se on desialylation of LDL and mitochondrial mutation are needed, which might provide better understand for the exact mechanism of Se effect on AS. Finally, our previous study demonstrated that BSA-SeNPs, at the dose of 50 μg Se/kg body weight/day, alleviated atherosclerotic lesions after oral administration for 8 or 12 weeks [26,27], but aggravated atherosclerotic lesions and exhibited toxicity to the liver and kidneys after long-term administration (24 weeks) in ApoE^-/-^ mice [55]. Therefore, in order to lower the toxicity of Se, CS-SeNPs or Na_2_SeO_3_, at the dose of 40 μg Se/kg body weight/day, were used in present study. However, whether CS-SeNPs or Na_2_SeO_3_ at the dose of 40 μg Se/kg body weight/day is still safe after long-term administration remains unknown. This problem should be solved in the future.

## 4. Materials and Methods

### 4.1. Materials

Na_2_SeO_3_ was purchased from Sigma-Aldrich. Chitosan (molecular weight about 26.3 kDa) was purchased from Regal Biology Technology Co., Ltd. (Shanghai, China). The kit for determining NO was purchased from Nanjing Jiancheng Institute of Biological Engineering (Nanjing, China), and the kits for determining MDA, T-AOC and GPx were purchased from Elabscience Biotechnology Co., Ltd. (Wuhan, China). ELISA kits for serum TNF-α and IL-6, First Strand cDNA Synthesis Kit, Trizol, SYBR Green PCR Master Mix kit and Dulbecco’s modified Eagle’s medium (DMEM) were purchased from Thermo Fisher Scientific. Fetal bovine serum (FBS) was purchased from Zhejiang Thianhang Biotechnology Co., Ltd. (Zhejiang, China). The enhanced chemiluminescence (ECL) kit, polyvinylidene fluoride (PVDF) membrane and all secondary antibodies were purchased from Millipore (Billerica, MA, USA). Antibodies against IκBα, p-IκBα, NF-κB p65 and p-p65 (Ser536) were purchased from Wanleibio (Shenyang, China). Antibody against β-actin and RIPA lysis buffer were purchased from Shanghai Beyotime Biotechnology Co., LTD (Shanghai, China). All chemicals were of analytical grade and used without further purification.

### 4.2. Preparation of Selenium Nanoparticles Decorated with Chitosan

The preparation of CS-SeNPs was carried out by reducing Na_2_SeO_3_ with ascorbic acid in the presence of chitosan, according to the method described in our previous work [30]. In briefly, chitosan (1% *w*/*w*) was added to double-distilled water under vigorous stirring, at first, and then Na_2_SeO_3_ (50 mM) and ascorbic acid (100 mM) were added into the mixture successively. At last, the reaction system was diluted to a final volume of 10 mL with double-distilled water, to ensure the final concentrations of Na_2_SeO_3_, ascorbic acid and chitosan were 5 mM, 20 mM and 3 μM, respectively. The reaction system was then stirred at room temperature for 1 h. CS-SeNPs were formed when the mixture changed from colorless to red. The as-synthesized CS-SeNPs products were collected by centrifugation with 4288× *g* at 4 °C for 20 min and washed three times with double-distilled water. Finally, the obtained CS-SeNPs was redissolved in double-distilled water and stored at 4 °C for further use.

### 4.3. Characterization of CS-SeNPs

The morphologies of the as-prepared CS-SeNPs were characterized by TEM (HT7700, HITACHI Co., Japan). The hydrodynamic size of the CS-SeNPs in aqueous was measured by DLS (LB-550, Horiba, Ltd., Tokyo, Japan). IR spectroscopy (Equinox 55, Brucker Optics, Bremen, Germany) in the range of 4000–500 cm^−1^ was test using the KBr-disk method. Zeta potential of CS-SeNPs was determined with Zetasizer Nano ZSP (Malvern Instrument, Malvern, Worcestershire, UK).

### 4.4. Animals

ApoE^-/-^ mice (C57BL/6 background), the most-used animal model of AS [56], were used in this study. Male ApoE^-/-^ mice (seven-week-old) of specific pathogen-free (SPF) were purchased from the Beijing HuaFuKang Bioscience Co. Ltd. (Beijing, China). All ApoE^-/-^ mice were fed with HFD (containing 0.15% cholesterol and 21% cream, obtained from Jiangsu Medicience Biomedicine Co. Ltd., Suzhou, China) after adaptive feeding with standard diet for 1 week. ApoE^-/-^ mice were randomly divided into the following three groups (*n* = 10 in each group): AS model group (Model), Na_2_SeO_3_ group and CS-SeNPs group. Ten sex- and age-matched wild type C57BL/6 mice purchased from Hubei Provincial Center for Disease Control and Prevention were used as reference control (WT). The CS-SeNPs and Na_2_SeO_3_ group was treated with CS-SeNPs and Na_2_SeO_3_ (40 μg Se/kg body weight) per day via an intragastric administration, respectively. The model and WT group were kept without any treatment except intragastric administration with normal saline per day. All mice were housed in plastic cages (five mice per cage) in a temperature-controlled environment (22 ± 2 °C, 12 h light/dark cycle) with free access to water and food. The body weights of the mice were observed and recorded weekly throughout the experiment period. After 10-week feeding, all mice were fasted overnight and euthanized by intraperitoneal injection of pentobarbital sodium. Blood and tissue samples were then obtained quickly for further analysis. All animal experiments were approved by the Institutional Laboratory Animal Ethics Committee of Huazhong University of Science and Technology (No. s1900).

### 4.5. Sampling

At the end of the experiment, blood samples were collected through transthoracic cardiocentesis. The serum was separated from blood immediately by centrifugation with 1070× *g* at 4 °C for 10 min and stored at aliquots at −80 °C for further analysis. Immediately after blood drainage, whole-body perfusion was performed with 0.01 M phosphate-buffered saline (PBS) through the heart to remove the residual blood, especially the blood in the aorta. The whole aorta was collected immediately. Some aortas were fixed in 4% paraformaldehyde for histopathological analysis, while others were preserved at −80 °C for other studies.

### 4.6. Atherosclerotic Lesion Analysis

To assess the level of atherosclerotic lesion, the whole aorta (from the aortic root to the iliac bifurcation) was opened longitudinally and the *en face* staining of Oil red O was performed according to the previous reports [56,57]. The images of the stained aorta were captured under a stereomicroscopy. The Oil red O-positively stained area was quantified using Image Pro Plus 6.0 software. The atherosclerotic lesion was determined as a percentage of staining area to total surface area of the whole aorta. For the analysis of atherosclerotic lesion in aortic root, the aortic root fixed in 4% paraformaldehyde was embedded in paraffin for sectioning, then paraffin sections were stained with HE for histopathological analysis.

### 4.7. Biochemical Analysis

The levels of serum NO, and MDA, and the activity of T-AOC and GPx were tested according to the manufacturer’s instructions using commercial kits. The contents of Se in liver were determined by inductively coupled plasma mass spectrometry (ICP-MS) [58]. The levels of serum TNF-α and IL-6 were tested according to the manufacturer’s instructions using commercial ELISA kits.

### 4.8. Immunofluorescence and Immunohistochemistry Analysis

Immunofluorescence staining for F4/80 was performed to characterize macrophages in aortic root. The aortic root cross sections were incubated with primary antibody against F4/80 (1:500) overnight at 4 °C. After washing with PBS for three times, the sections were incubated with a fluorescence-labeled secondary antibody (goat anti-rat, 1:300) at room temperature for 50 min. The samples were then stained with DAPI for 3 min, followed by washing with PBS for three times. The red reaction product for F4/80 and blue reaction product for DAPI were obtained and at least three stained sections images were captured with inverted fluorescence microscopy.

Immunohistochemical staining for α-SMA was performed to characterize VSMCs in aortic root. The aortic root cross sections were incubated with primary antibody against α-SMA (1:200) overnight at 4 °C. After washing with PBS for three times, the sections were incubated with a horse radish peroxidase (HRP) labeled secondary antibody (goat anti-mice, 1:200) at room temperature for 50 min. The reaction products were revealed by immersing the slides in diaminobenzidine tetrachloride to give a brown reaction product. At least three stained sections images were captured with inverted fluorescence microscopy. All reagents used in immunofluorescence and immuno-histochemistry analysis were purchased from Wuhan Servicebio technology Co., LTD (China).

### 4.9. Cell Culture and Treatment

The human umbilical vein EC line, EA.hy926, was purchased from Cell Resource Center of Shanghai Institute of Life Sciences, Chinese Academy of Sciences. ECs were cultured in DMEM with 15% FBS and antibiotic solution at 37 °C in 5% CO_2_. To assess the effect of CS-SeNPs and Na_2_SeO_3_ on LPS-induced cellular injury, inflammation and endothelial dysfunction, ECs were divided into the following four groups for different treatment: control group (Con), LPS-induced group (LPS), CS-SeNPs-pretreated group (CS-SeNPs), and Na_2_SeO_3_-pretreated group (Na_2_SeO_3_). The control group were cultured normally without any treatment. LPS-induced group were stimulated with 1 μg/mL LPS for 12 h. The CS-SeNPs- and Na_2_SeO_3_-pretreated groups were pretreated with 0.5 μM CS-SeNPs or Na_2_SeO_3_, respectively, for 12 h and then exposed to 1 μg/mL LPS for another 12 h.

### 4.10. Determination of Gene Expression by qPCR

The mRNA expression levels of IL-6, IL-1β, TNF-α, MIP-1α, MCP-1, E-selectin, VCAM-1, ICAM-1 and iNOS in aorta or ECs were measured by qPCR. In brief, total RNA was isolated from homogenizing aorta tissues or ECs using Trizol reagent and reverse-transcribed into cDNA. Total RNA was purified by chloroform, isopropanol and 75% alcohol, and the purity of total RNA (260/280 nm ratio) was determined using a microplate analyzer (Varioskan LUX, Thermo Scientific, Waltham, MA, USA). The reverse transcription was performed when the ratio of 260/280 nm was more than 1.8. SYBR Green PCR Master Mix kit was used for PCR amplification performed on an ABI StepOne™ Real-Time PCR System (Applied Biosystems, Foster, CA, USA). Both reverse transcription and PCR amplification were done according to the vendors’ protocols. All primers sequences used in the detection for aorta and ECs were listed in Table S2 and Table S3, respectively. The 2^−ΔΔCT^ method was used for quantification in StepOne Plus Software v2.3 with β-actin or GADPH gene as an internal control [59].

### 4.11. Western Blot Analysis

Total protein was extracted from ECs using RIPA lysis buffer and concentrations were determined by the Lowry method [60]. Proteins were denatured and prepared for Western blot analysis. Protein samples were separated by sodium dodecyl sulfate polyacrylamide gel electrophoresis (SDS-PAGE) and transferred to a PVDF membrane. After blocking with 3% bovine serum albumin at room temperature for 2 h, the membranes were incubated with the indicated primary antibody overnight at 4 °C, followed by an incubation with a horseradish peroxidase conjugated secondary antibody at room temperature for 1.5 h. The blots were visualized using an ECL kit and the relative expressions of target proteins were quantified using Tanon 5200 MultiImage System (Tanon, Shanghai, China).

### 4.12. Statistical Analysis

The representative results at least three independent experiments are expressed as mean ± SD. Differences among groups was analyzed by One-way ANOVA analysis. The differences between groups were compared by Tamhane’s T2 test when the variance was not uniform. A difference with *p* < 0.05 was considered significant. All statistical analyses were performed using SPSS 22.0 software (IBM, Armonk, NY, USA).

## 5. Conclusions

**Figure 11:**
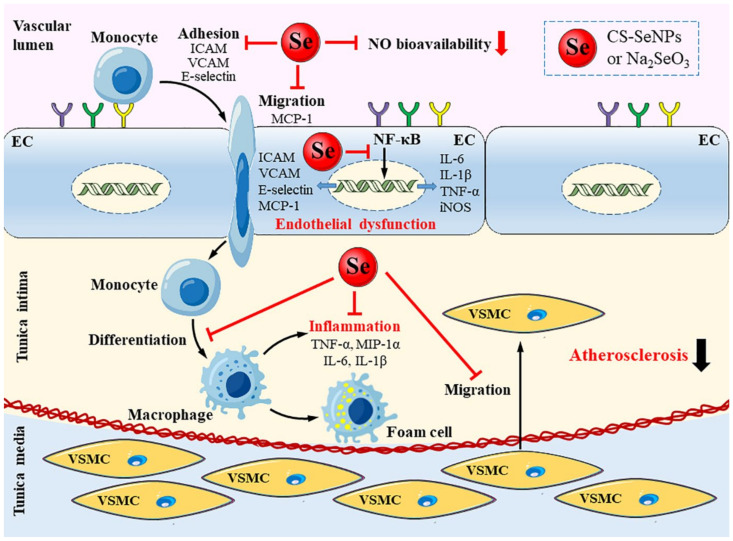
Schematic diagram for the alleviated effect of CS-SeNPs and Na_2_SeO_3_ on endothelial dysfunction, inflammation and atherosclerotic lesions. CS-SeNPs and Na_2_SeO_3_ suppressed endothelial NF-κB activation and subsequently target gene expression including adhesion molecules VCAM-1, E-selectin and ICAM-1, chemokine MCP-1, cytokines TNF-α, IL-6 and IL-1β, pro-inflammatory molecule iNOS. Consequently, macrophages recruitment in the intima, the expression of macrophage-derived TNF-α, MIP-1α, IL-6, IL-1β, VSMC migration and atherosclerotic lesion formation are reduced.

In summary, our results illustrated that both CS-SeNPs and Na_2_SeO_3_ at dose of 40 μg/kg body weight/day significantly ameliorated early atherosclerotic lesions in HFD fed ApoE^-/-^ mice after oral administration for 10 weeks. The underly mechanisms might including alleviating endothelial dysfunction and inflammatory response (Figure 11). CS-SeNPs and Na_2_SeO_3_ suppressed endothelial NF-κB activation and, subsequently, target gene expression, including adhesion molecules VCAM-1, E-selectin and ICAM-1, chemokine MCP-1, cytokines TNF-α, IL-6 and IL-1β, pro-inflammatory molecule iNOS, leading to decreased endothelial dysfunction and inflammatory response. Overall, although more detailed investigations are still awaited to understand the pharmacokinetics and pharmacodynamics, both CS-SeNPs and Na_2_SeO_3_ played a positive role in the prevention of AS and CS-SeNPs showed a similar effect to Na_2_SeO_3_. Considering the low toxicity, SeNPs may have a greater potential promise than Na_2_SeO_3_. The present study provides more insights into the mechanisms of Se against AS and further highlight the potential of Se as a therapeutic strategy for AS.

## Figures and Tables

**Table 1:** Effect of CS-SeNPs and Na_2_SeO_3_ on serum T-AOC, GPx activity and MDA content.

Group	MDA (nmol/mL)	T-AOC (U/mL)	GPx (U/mL)
WT	1.10 ± 0.19	5.09 ± 0.78	260.60 ± 59.53
Model	8.93 ± 0.57 ^###^	1.76 ± 0.33 ^###^	154.12 ± 33.55 ^###^
Na_2_SeO_3_	7.64 ± 0.46 **	2.29 ± 0.35 *	280.48 ± 54.11 ***
CS-Se	8.46 ± 1.32	2.78 ± 0.51 **	191.59 ± 33.45 *

Note: The results were expressed as mean ± SD (*n* = 8–9). ^###^ *p* < 0.001, compared with the WT group. * *p* < 0.05, ** *p* < 0.01, *** *p* < 0.001, compared with the AS model group.

## Supplementary Materials

The following are available online at https://www.mdpi.com/article/10.3390/ijms222111612/s1.

## Abbreviations

ApoE^-/-^, apolipoprotein E-deficient; AS, atherosclerosis; CS, chitosan; CS-SeNPs, SeNPs stabilized with chitosan; CVD, cardiovascular diseases; DLS, dynamic light scattering; ECs, endothelial cells; GPx, glutathione peroxidase; HE, hematoxylin-eosin; HFD, a high-fat diet; ICAM-1, intercellular adhesion molecule-1; IL-6, interleukin-6; IL-1β, interleukin-1β; iNOS, inducible nitric oxide synthase; IR, infrared; LDL, low-density lipoprotein; LPS, lipopolysaccharide; MCP-1, monocyte chemoattractant protein-1; MDA, malondialdehyde; MIP-1α, macrophage inflammatory ptotein-1α; NF-κB, nuclear factor-κB; NO, nitric oxide; qPCR, real-time quantitative polymerase chain reaction; Se, selenium; SeMet, selenomethionine; SeMSC, methylselenocysteine; SeNPs, Se nanoparticles; TEM, transmission electron microscope; T-AOC, total antioxidant capacity; TNF-α, tumor-necrosis factor-α; TrxR, thioredoxin reductase; VCAM-1, vascular cell adhesion molecule-1; VSMCs, vascular smooth muscle cells; XPS, X-ray photoelectron spectra; XRD, X-ray diffraction.
