# Supplementary material for: Comparison of Selenium Nanoparticles and Sodium Selenite on the Alleviation of Early Atherosclerosis by Inhibiting Endothelial Dysfunction and Inflammation in Apolipoprotein E-Deficient Mice
Source: Int J Mol Sci. 2021 Oct 27;22(21):11612. doi: 10.3390/ijms222111612 (PMC8583811; doi:10.3390/ijms222111612)
Supplement: Supplementary file 1 [file ijms-22-11612-s001.zip › ijms-1419221-supplementary.pdf]

Supplementary Information

Comparison of Selenium Nanoparticles and Sodium Selenite on the Alleviation of Early Atherosclerosis by Inhibiting Endothelial Dysfunction and Inflammation in Apolipoprotein E-Deficient Mice

Xiao Junying, Li Na, Xiao Shengze, Wu Yuzhou and Liu Hongmei

Table S1: Average food intake (g/day/mouse). The results were expressed as mean ± SD.

Age (week)	Model	Na_2SeO_3	CS-SeNPs
9	2.84± 0.32	2.75± 0.18	2.93± 0.16
10	3.26±0.11	3.07±0.16	3.12±0.17
11	3.53±0.16	3.28±0.18	3.12±0.21
12	3.21±0.18	2.71±0.18	2.79±0.18
13	3.39±0.16	2.98±0.12	2.90±0.29
14	3.04±0.13	2.76±0.11	3.08±0.17
15	2.84±0.11	2.37±0.13	2.49±0.13
16	3.42±0.40	2.91±0.16	3.02±0.11
17	3.15±0.20	2.65±0.15	2.61±0.19
18	2.89±0.18	2.45±0.15	2.41±0.27

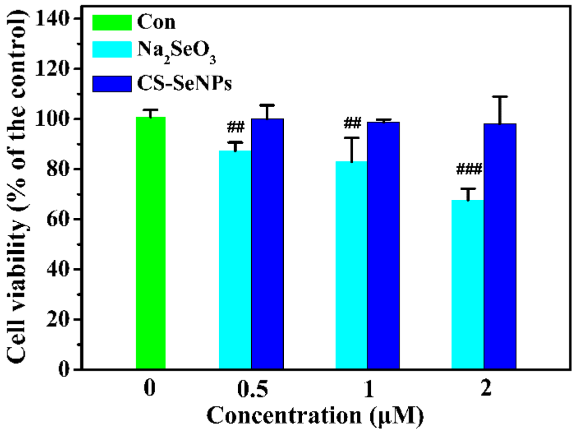

Figure S1: Effect of CS-SeNPs and Na_2SeO_3 on the viability of human endothelial cell line EA.hy926. Cells were treated with indicated concentrations of CS-SeNPs or Na_2SeO_3 for 24 h. The cell viability was determined using the MTT assay (mean ± SD, n = 4). $^{\#\#}$ $P < 0.01$, $^{\#\#\#}$ $P < 0.001$, compared with control cells.

Table S2: Primers sequences used for the detection of related gene expression in the aorta of ApoE$^{-/-}$ mice by qPCR

Gene	Primer sequence 5` to 3`	Product length (bp)
TNF-α	F: ACCCTCACACTCAGATCATCTT<br>R: GGTTGTCT TGAGATCCATG	125
IL-6	F: CCTTCCTACCCCAATTTCCAA<br>R: AGATGAATTGGATGGTCTTGGTC	89
IL-1β	F: TGCCACCTTTTGACAGTGATG<br>R: GAGTGATACTGCCTGCCTGA	125
MIP-1α	F: CCTCTGTCACCTGCTCAACA<br>R: GATGAATTGGCGTGGAATCT	163
MCP-1	F: CCACTCACCTGCTGCTACTCAT<br>R: TGGTGATCCTCTTGTAGCTCTCC	76
E-selectin	F: GTGAAGCTTTGACCCACCCT<br>R: ATTCTGAGCTCCAACTCGCC	125
VCAM-1	F: CTGGGAAGCTGGAACGAAGT<br>R: GCCAAACACTTGACCGTGAC	115
ICAM-1	F: GTGATGCTCAGGTATCCATCCA<br>R: CACAGTTCTCAAAGCACAGCG	213
β-actin	F: GATCAAGATCATTGCTCCTCCT<br>R: AGGGTGTAAAACGCAGCTCA	183

Table S3: Primers sequences used for the detection of related gene expression in human ECs by qPCR

Gene	Primer sequence 5` to 3`	Product length (bp)
TNF-α	F: GTGACAAGCCTGTAGCCCATGTT<br>R: TTATCTCTCAGCTCCACGCCATT	112
IL-6	F: ACTCACCTCTTCAGAACGAATTG<br>R: CCATCTTTGGAAGGTTCAGGTTG	149
IL-1β	F: ATGATGGCTTATTACAGTGGCAA<br>R: GTCGGAGATTCGTAGCTGGA	132
MCP-1	F: GCTCATAGCAGCCACCTTCATTC<br>R: GGACACTTGCTGCTGGTGATTC	147
iNOS	F: GATCAAAAACTGGGGCAGCG<br>R: CTCATCTGGAGGGGTAGGCT	157
E-selectin	F: GCACAGCCTTGTCCAACC<br>R: ACCTCACCAAACCCTTCG	228
VCAM-1	F: GGTATCTGCATCGGGCCTC<br>R: TAAAAGCTTGAGAAGCTGCAAACA	193
ICAM-1	F: GGAACAACCGGAAGGTGTATG<br>R: TGCCAGTTCCACCCGTTCT	140
GADPH	F: CACCAGGGCTGCTTTTAACTCTGGTA<br>R: CCTTGACGGTGCCATGGAATTTGC	131
